# Whole-Genome Sequencing of the World’s Oldest People

**DOI:** 10.1371/journal.pone.0112430

**Published:** 2014-11-12

**Authors:** Hinco J. Gierman, Kristen Fortney, Jared C. Roach, Natalie S. Coles, Hong Li, Gustavo Glusman, Glenn J. Markov, Justin D. Smith, Leroy Hood, L. Stephen Coles, Stuart K. Kim

**Affiliations:** 1 Depts. of Developmental Biology and Genetics, Stanford University, Stanford, CA, United States of America; 2 Institute for Systems Biology, Seattle, WA, United States of America; 3 Gerontology Research Group, Los Angeles, CA, United States of America; 4 David Geffen School of Medicine, University of California Los Angeles, Los Angeles, CA, United States of America; UCL Institute of Neurology, United Kingdom

## Abstract

Supercentenarians (110 years or older) are the world’s oldest people. Seventy four are alive worldwide, with twenty two in the United States. We performed whole-genome sequencing on 17 supercentenarians to explore the genetic basis underlying extreme human longevity. We found no significant evidence of enrichment for a single rare protein-altering variant or for a gene harboring different rare protein altering variants in supercentenarian compared to control genomes. We followed up on the gene most enriched for rare protein-altering variants in our cohort of supercentenarians, TSHZ3, by sequencing it in a second cohort of 99 long-lived individuals but did not find a significant enrichment. The genome of one supercentenarian had a pathogenic mutation in DSC2, known to predispose to arrhythmogenic right ventricular cardiomyopathy, which is recommended to be reported to this individual as an incidental finding according to a recent position statement by the American College of Medical Genetics and Genomics. Even with this pathogenic mutation, the proband lived to over 110 years. The entire list of rare protein-altering variants and DNA sequence of all 17 supercentenarian genomes is available as a resource to assist the discovery of the genetic basis of extreme longevity in future studies.

## Introduction

Supercentenarians are the world’s oldest people, living beyond 110 years of age [1]. As would be expected for people that reach this age, supercentenarians have escaped many age-related diseases [2]–[5]. For example, there is a 19% lifetime incidence of cancer in centenarians compared to 49% in the normal population [6]. Similarly, supercentenarians have a lower incidence of cardiovascular disease and stroke than controls [5].

The genetic component of human lifespan based on twin studies has been estimated to be around 20–30 percent in the normal population [7], but higher in long-lived families [8]–[10]. Furthermore, siblings, parents, and offspring of centenarians also live well beyond average [11], [12]. Lifestyle choices in terms of smoking, alcohol consumption, exercise, or diet does not appear to differ between centenarians and controls [13]. Taken together, these findings provide ample evidence that extreme longevity has a genetic component.

Several gene association studies have compared cohorts of long-lived subjects to controls. Analysis of candidate genes has shown that polymorphisms in the Insulin-like Growth Factor 1 Receptor gene (IGF1R) and the FOXO3 transcription factor gene are associated with extreme longevity [14], [15]. Genome-wide association studies have shown that the ApoE4 haplotype is depleted in centenarians [16]–[18]. Sebastiani et al. compiled a list of 281 independent single-nucleotide polymorphisms (SNPs) that showed strong associations with extreme longevity (though none were genome-wide significant except for an ApoE SNP) [17]. They then showed that a genetic signature that combines information from these 281 SNPs is predictive for extreme longevity, indicating that at least some of these SNPs are truly associated with longevity. However, specific variants associated with longevity have not yet been identified [18], [19].

More recently, studies have begun to use whole-exome sequencing and whole-genome sequencing (WGS) of centenarians to find variants associated with extreme longevity [19]–[21]. Ye et al. compared the genome sequence of a pair of 100-year-old twins to a pair of 40-year-old twins and found no evidence of accumulation of somatic mutations during aging [20]. By sequencing blood cells of a supercentenarian, Holstege et al. first identified somatic mutations and then used this information to infer clonal lineages in hematopoietic stem cells. They found that white blood cells in this individual were derived from only two clones of hematopoietic stem cells [21].

Here, we have sequenced the genomes of 17 supercentenarians. We limited the majority of our analyses to the thirteen genomes from Caucasian females. From this small sample size, we were unable to find rare protein-altering variants significantly associated with extreme longevity. However, we did find that one supercentenarian carries a pathogenic variant associated with arrhythmogenic right ventricular cardiomyopathy (ARVC), which had little or no effect on his/her health as this person lived over 110 years.

## Materials and Methods

### Ethics Statement, Supercentenarian Recruitment and Age Validation

Supercentenarian subjects, their family members, or their caretakers provided written informed consent. The study was approved by the Stanford University Institutional Review Board (IRB-19119) and by the Western Institutional Review Board (WIRB protocol #20101350). Supercentenarians were considered validated (i.e., 110 years or older) if they possessed each of the following documents: (1) A birth certificate, a baptismal certificate, or Census Record dating back to the original time of birth; (2) A marriage certificate in the case of married women not using their maiden names; (3) a current government-issued photo ID, such as a driver’s license or passport. Supercentenarian health status and medical history for major age-related diseases were based on interviews conducted with subjects and/or their caretakers.

### DNA Isolation, PCR and Sanger Sequencing

Whole-blood samples were drawn into PAXgene (Qiagen) blood tubes from which high molecular weight DNA was isolated. DNA samples were quantified using a dsDNA Broad-Range Assay on a Qubit Fluorometer (Life Technologies) and checked for size and degradation on an agarose gel. For Sanger sequencing, samples were amplified by nested PCR and variants were validated by forward and reverse reads. Primers were designed with Primer3 [22] and 10 ng was amplified with Phusion High-Fidelity Polymerase (Thermo Scientific). PCR bands were either column-purified or cut out from an agarose gel and purified with a Qiaquick Gel Extraction kit (Qiagen). PCR product was Sanger sequenced at Sequetech, Inc. Reads were trimmed by 10 bp at the 5′ end and at a 0.01 error probability limit and then aligned to the human genome reference sequence build GRCh37 (hg19) using Geneious software. For sequencing of TSHZ3 in the Georgia Centenarian Study samples, all coding regions were sequenced except the first 13 amino acids (i.e., exon 1). None of the rare protein altering variants found in the 13 supercentenarians or the 4,300 NHBLI controls were located in exon 1. All experiments were performed according to manufacturer’s protocol unless otherwise indicated.

### Ancestry and Relatedness

Principal component analysis (PCA) of ancestry was done by analyzing the intersection of all genotyped SNPs from 1184 individuals from 12 different populations from HapMap Phase 3 [23] and the 17 supercentenarians. Only bi-allelic SNPs that had at least one non-reference allele in the 17 supercentenarians were used, resulting in a subset of 1.2 million SNPs. Genome-wide Complex Trait Analysis (GCTA) software was used to perform the PCA [24]. All pairs of 17 supercentenarians were tested for relatedness using Estimation of Recent Shared Ancestry (ERSA) [25], [26].

### Whole-Genome Sequencing and Analysis Pipelines

All DNA samples were submitted for WGS to 40x coverage by Complete Genomics, Inc. (CGI). Standard protocols were used to map reads and call variants using CGI pipeline 2.0.2 [27]. To analyze variants, we first produced a cross-reference matrix out of CGI variant files using custom Perl scripts [28] and the CGI command line tool CGAtools (listvar, testvar). To reduce platform errors and biases, we removed any variant with >50% double no-call rate in a control set of public genomes sequenced on the same CGI platform. We used 54 of the unrelated HapMap genomes (for variant analysis) or the 34 PGP genomes (for the RVT1 burden test). The 54 HapMap Genomes were obtained as part of the public CGI Diversity panel of 69 and the 34 PGP genomes were obtained from the Personal Genome Project [29]. The baseline characteristics of the 34 PGP genomes are listed in Table S1. Next, we used ANNOVAR and its build GRCh37 (hg19) database files [30] and custom scripts to annotate protein-altering variants: missense, frameshift, non-frameshift indels, stop-gain, stop-loss, and splice-site disruption. Splice-site variants were those disrupting the canonical splice-donor (GU) or splice-acceptor (AG) site of the RefSeq sequence. To test for enrichment of a rare protein-altering variant, we used the 379 European individuals from the 1000Genome (1000G EUR) Project Phase 1 (April 2012) build database as controls [31]. We included all protein-altering variants and did not require missense SNPs to be predicted as damaging by, e.g., SIFT or PolyPhen-2.

To filter out common variants, we used dbSNP version 131 [32]. This version was released on February 2010, and lacks most low-frequency variants deposited by large consortia like NHLBI and 1000G in later versions. Rare variants were tested for enrichment in cases (13 Caucasian female supercentenarians) vs. controls (379 European individuals from the 1000G Project) using Fisher’s Exact Test. We repeated our analysis with reduced stringency by lowering the quality score threshold, but we did not see any significantly enriched variant or gene. Consistent with previous reports, Sanger sequencing of candidate rare protein-altering variants from WGS showed that 30 percent were likely sequencing errors [33], [34].

Next, we applied a collapsing test to determine if any gene showed an enrichment of rare protein-altering variants in supercentenarian vs. control genomes. We started with the set of protein-altering variants in autosomal RefSeq genes observed in supercentenarians and controls (34 Caucasians from the PGP), and filtered to retain only rare variants with a minor allele frequency (MAF) <1.5% in 1000G EUR, and with an empirical MAF<10% in our samples. For each gene, we computed the RVT1 statistic [35] to determine whether the burden of mutations differed in supercentenarians and controls using R scripts [36]. RVT1 performs a logistic regression to model phenotype (case/control status) as a function of the proportion of rare variants seen in each genome. We repeated our burden test using a 5% instead of 1.5% as the 1000G EUR MAF cutoff, and again saw no significantly enriched gene. For the recessive model test, we compared all subjects having two or more variants per gene and scored significance using Fisher’s Exact test.

### Cohorts used to follow-up TSHZ3 variants

Samples from the Georgia Centenarian Study [37] were obtained from Coriell as DNA samples (Coriell ID: AGPLONG3). All Caucasian samples (n = 100) were analyzed and used for PCR and Sanger sequencing as described above. Two of our supercentenarians had previously participated in the Georgia Centenarian Study; their samples were identified by genotyping and removed from the cohort (NG18205, NG20051). In addition, we checked that none of the other supercentenarians with a protein-altering variant in TSHZ3 was present in the Georgia cohort by Sanger sequencing several loci in the Georgia cohort. We added a female Caucasian centenarian sample from our own study (age 100), bringing the total to 99. For controls, we used exome data for 4,300 Caucasians obtained from the NHLBI Exome Variant Server [38].

### Analysis of Pathogenic Variants

We used the recently published list from the American College of Medical Genetics and Genomics (ACMG) of potentially lethal pathogenic variants in 56 genes recommended for reporting to subjects [39]. All 17 supercentenarian genomes were annotated as described above, except without filtering for common variants. ClinVar and Human Gene Mutation Database (HGMD) were used to identify known pathogenic variants in the supercentenarian genomes in all 56 genes identified by the ACMG [40], [41]. Besides the known pathogenic variants, new variants can be expected to be pathogenic in 45 of the 56 genes if the new variant clearly strongly reduces or eliminates protein function, such as frameshift, stop-gain, stop-loss, or splice-site mutations [42]. Any variant suggested to be benign based on annotation in ClinVar or HGMD was removed. The scoring of variants as either pathogenic or benign was also checked using Locus Specific Databases (LSDB). Pathogenic annotation of the c.631-2A>G mutation in DSC2 was confirmed in the Arrhythmogenic Right Ventricular Dysplasia/Cardiomyopathy (ARVD/C) database [43], which is part of the Leiden Open Variation Database [44].

### Data Access

Upon acceptance for publication, the complete genome sequence for the 17 supercentenarians will be deposited in dbGAP and Google Genomics.

## Results

### The Supercentenarian Cohort

We recruited 17 supercentenarians and validated their age of 110 years or greater (see Methods). Their mean age at time of death was 112 years and the subject that lived the longest died at the age of 116 years. At the time of her death, she was the world’s oldest person and remains in the top ten of oldest people in recorded history [45]. We determined the medical history and health status of supercentenarians at the time of enrollment by interviewing them, their family, and caretakers. Many of the supercentenarians were cognitively and physically functional to a high degree well into old age. For example, one of our subjects worked as a pediatrician until the age of 103. Another subject drove a car until the age of 107. Table 1 gives an indication of some of the aspects of the supercentenarian health at the time of blood draw.

**Table 1:** Characteristics of supercentenarians.

Age	Age atDraw	Sex	Race	Major Age-relatedDiseases	Hearing	Vision	Dental	Communi-cation	Mobility
116	114	F	CAU	None	••	••	•••	•••	•
114	110	F	HIS	None	••	•	•	••	••
114	112	F	CAU	None	•	•	•	•	•
114	112	F	CAU	None	•	•	•	•	••
114	114	F	CAU	None	•	•	•	•	•
114	110	F	HIS	None	••	••	•••	••	•••
113	111	F	CAU	None	•••	•••	•••	••	••
113	112	F	CAU	None	•	•	•	••	••
113	113	F	AA	None	•••	•••	•••	••	••
112	110	F	CAU	None	••	••	•••	••	••
111	110	F	CAU	Alzheimer’s	•	•	•	•	•
111	110	F	CAU	None	•	•••	•	••	••
111	110	F	CAU	None	•	•	•	••	••
111	110	F	CAU	None	••	•••	•••	•••	•••
111	110	M	CAU	Cancer	•••	•••	•••	••	•••
111	111	F	CAU	None	•	•	•	••	••
110	110	F	CAU	None	•	•	•	••	••

Age is age at death or last reported age alive. Age at (blood) draw was validated as described in methods. Sex is female (F) or male (M). Race (or ethnicity) is Caucasian (CAU), Hispanic (HIS) or African-American (AA). Major age-related diseases were known events of cancer, cardiovascular disease, stroke, Alzheimer’s or type 2 diabetes at blood draw (i.e. enrollment). Functional status is indicated as: ••• (good), •• (moderate) or • (poor). Hearing: ••• good in both ears; •• impaired in one, good in other ear; • impaired in both ears. Vision: ••• could read newspaper; •• could watch television; • could do neither. Teeth: ••• had teeth of their own; • no teeth of their own. Communication: ••• talked independently and coherently; •• slow speech, needed interpreter; • incoherent or no communication. Mobility: ••• could walk; •• uses wheelchair; • bed confined.

Among the 17 supercentenarians, at least one subject had a previous case of cancer and one was diagnosed with Alzheimer’s disease. To the best of our knowledge, none of the supercentenarians were known to have cardiovascular disease, stroke or diabetes at the time of enrollment. In contrast, people in the US at age 85 often have had at least one major age-related disease. For example, 45 percent of 85-year olds have been diagnosed with cancer and 35 percent have had an incidence of cardiovascular disease [5]. The low rate of disease in our cohort of supercentenarians is consistent with previous reports showing that supercentenarians delay or escape most age-related diseases [5].

We isolated DNA from whole blood and sent the samples to Complete Genomics for WGS. Samples were sequenced to a read depth of 40x, and 94.1% of the genomes and 94.8% of the exomes had a read depth of at least 20x (Figure S1). To confirm the self-reported ancestry of all subjects, we performed a Principal Component Analysis (PCA) on the genomes of our 17 supercentenarians and that of 1184 HapMap individuals with known ancestry to serve as controls (Figure S2). This analysis confirmed that 14 supercentenarians were of European ancestry, one was African American, and two were Hispanic. To prevent confounding our analyses due to differences in race or sex, we used only supercentenarian genomes that were both Caucasian and female for our main analyses. This left us 13 genomes for the main analysis with one male, two Hispanic, and one African-American genome reserved for follow-up analyses.

Next, we checked the genomes of our supercentenarians for unknown relatedness to each other, as any close relationship would confound analyses for enrichment of shared rare variants. We checked for shared regions of identity-by-descent using Maximum-likelihood Estimation of Recent Shared Ancestry [25], [26]. The results indicated that none of the 17 supercentenarians were within five degrees of relationship of any other supercentenarian, which means that at least 97 percent of any of the supercentenarian genomes was not identical-by-descent to any of the other supercentenarian genomes.

### Are Supercentenarians Enriched for a Rare Protein-Altering Variant?

**Figure 1:**
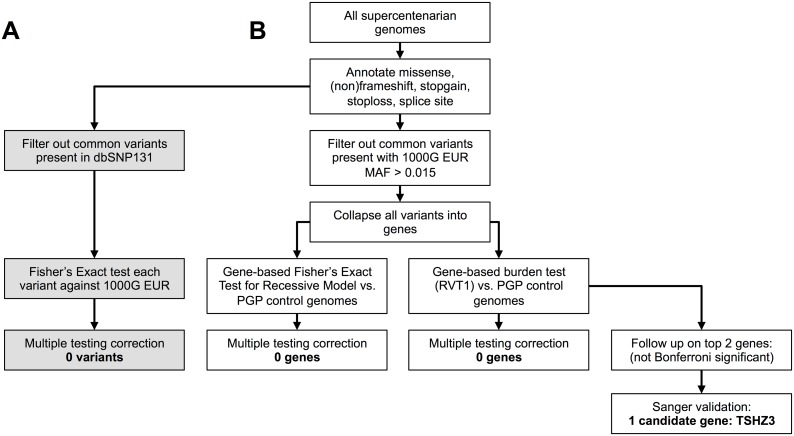
Pipeline to test supercentenarians for enrichment of rare protein-altering variants or genes harboring them. All female Caucasian supercentenarian genomes were annotated for protein-altering variants. (A) To test for enrichment of a single variant, we filtered against dbSNP131 and compared each remaining rare protein-altering variant against 1000G EUR. No single variant was significantly enriched. (B) To test for enrichment of a gene with rare protein-altering variants, we collapsed all variants in to their respective genes and filtered against 1000G EUR (MAF<0.015). We tested for enrichment against 34 control genomes from PGP using the RVT1 burden test or a gene-based Fisher’s Exact (for recessive model). No gene was significantly enriched for rare protein-altering variants in supercentenarians. We then Sanger validated TSHZ3 as the best candidate from our burden-test for follow-up.

For people born around 1900, the odds of living to 110 are estimated to be less than 10^−5^ per birth [46], hence we assume that any genetic variant that contributes strongly to extreme longevity would also be rare. One possibility is that a specific mutation could alter the protein-coding region in a gene and confer a significant increase in longevity. Such a mutation could act in a dominant or recessive fashion, and might be shared by a significant fraction of the supercentenarian genomes but not by control genomes. We created a computational pipeline to determine whether our supercentenarian genomes are enriched for such a variant compared to controls (Figure 1). We annotated the variants in all of the female Caucasian genomes and retained those predicted to alter a protein. The polymorphism could be a single nucleotide polymorphism (SNP) or an insertion/deletion (Indel). The polymorphism could change the protein-coding sequence by causing a missense, frameshift, non-frameshift indel, nonsense (i.e., stop-gain), stop-loss, or splice-site disruption (Table S2). To identify rare variants, we filtered out common variants by removing any variant present in the public database dbSNP build 131. We then compared the frequency of the rare protein-altering variants in the supercentenarian genomes with that in the 379 European individuals in the 1000Genomes Project (1000G EUR) using a Fisher’s Exact Test. In total, there were 13,892 rare protein-altering variants screened in the supercentenarian genomes. To adjust for multiple hypothesis testing, we applied a Bonferroni correction using a threshold of P<0.05/13,892 = 3.6×10^−6^. A variant that was present in four supercentenarian genomes but absent in all genomes in 1000G EUR would have a P-value of 7.4×10^−07^ and would have been detected by our method. Using high quality sequence calls, preliminary analysis suggested that one novel variant was shared by three supercentenarian subjects but not by the control genomes; however, Sanger sequencing subsequently showed that this was a sequencing error in the supercentenarian data. To increase our sensitivity for finding a longevity variant, we repeated the analysis including low quality calls. This yielded three additional novel variants in the supercentenarian genomes. However, Sanger sequencing showed that each of the three variants was a sequencing error. Even though the overall error rates for SNPs in WGS data (>40x coverage) are under 1% [27], the process of screening for apparent rare protein-altering variants also enriches for sequencing errors [33]. Therefore, we conclude that we found no evidence for a statistically significant enrichment of a specific protein-altering variant in the female Caucasian supercentenarian genomes compared with controls. Table S2 contains a list of the rare coding variants found in our 17 supercentenarian and 34 PGP control genomes.

### Are Supercentenarians Enriched for a Gene with Rare Protein-Altering Variants?

Another possibility is that there may be a gene that confers extreme longevity when it is altered by any one of a number of protein alterations. Many of the supercentenarians may carry variants in the same gene, but the variant in each supercentenarian may be different. The variants could act in a dominant fashion and affect only one of the two alleles. Or else they could act in a recessive fashion such that both alleles would be affected, either with the same variant (homozygous) or with different mutations in each allele (compound heterozygous). Therefore, we asked whether any of the genes in the female Caucasian supercentenarian genomes was enriched for harboring rare protein-altering variants (either one or two copies) when compared to control genomes. Although the 1000G are a large group of controls, they cannot be used for a gene-based test as only the frequency of each variant is known, and not the individual genotypes. Therefore, as controls we used WGS of 34 Caucasian individuals (ages 21–79) from the Personal Genome Project (PGP) that were sequenced on the same platform as the supercentenarians [29].

We created a pipeline that used the annotated supercentenarian and PGP genomes from the previous analyses as input (see also Figure 1). Next, we filtered out common variants, which we defined as having a minor allele frequency of 1.5% or higher in the 1000G EUR (i.e., Caucasian populations in the 1000G). For each gene and each genome, we counted the number of rare protein-altering variants. We then computed the RVT1 statistic [35] to determine whether any gene showed a different burden of variants in supercentenarians vs. controls.

There were 10,508 genes with at least one rare, protein-altering variant in controls or supercentenarians. We used a Bonferroni threshold of P<0.05/10,508 = 4.7×10^−06^ to correct for multiple hypothesis testing. We were thus powered to detect genes altered in seven supercentenarians, if the gene harbored no alleles in any of the 34 controls. None of the genes showed a genome-wide significant enrichment using the Bonferroni threshold (Table S3). Furthermore, we performed pathway analysis but failed to find a genetic pathway that showed a significant difference between supercentenarians and controls; specifically, we performed Gene Set Enrichment Analysis [47] using the results of the gene burden test, but no KEGG [48] pathway or Gene Ontology [49] category was significant at a false discovery rate <25%. To increase our sensitivity, we repeated our analyses including low-quality calls. This time, two genes initially appeared to be enriched for rare protein-altering alleles in the supercentenarian genomes, but Sanger sequencing showed that many of the variants were WGS errors.

We also specifically tested a recessive model for a gene conferring exceptional longevity, in which both alleles of a gene might harbor mutations. Supercentenarians would be enriched for carrying two or more different variants in such a gene (consistent with compound heterozygosity, if the mutations are out of phase), but controls would only carry zero or one mutation, but not two. The RVT1 test performs a logistic regression on the proportion of rare variants and hence might detect a bias in supercentenarians (two alleles in the gene) vs. the controls (one or zero alleles). But the RVT1 test was not specifically designed to compare the number of compound heterozygous cases and controls. We performed a gene-based test to compare the number of cases and controls carrying at least two variants in the same gene applying Fisher’s Exact Test to compute P values. We found that no gene was significantly enriched for two or more mutations after multiple testing correction (Table S4).

Although none of the genes showed a significant enrichment in the female Caucasian supercentenarian genomes, we nevertheless decided to follow up on the top three genes from the RVT1 burden test: TSHZ3, NAB2, and SCN11A (each with nominal P = 4.3×10^−4^). For SCN11A, three control genomes contained rare protein-altering variants with minor allele frequencies below 0.05 (but above 0.01). This result weakens the distinction between the supercentenarian genomes and the control genomes, and thus this gene was discarded from further analysis. NAB2 was discarded when Sanger sequencing showed that two out of four variants were sequencing errors. For TSHZ3, Sanger sequencing validated all four protein-altering variants, and this gene was chosen as a candidate for follow-up experiments.

**Figure 2:**
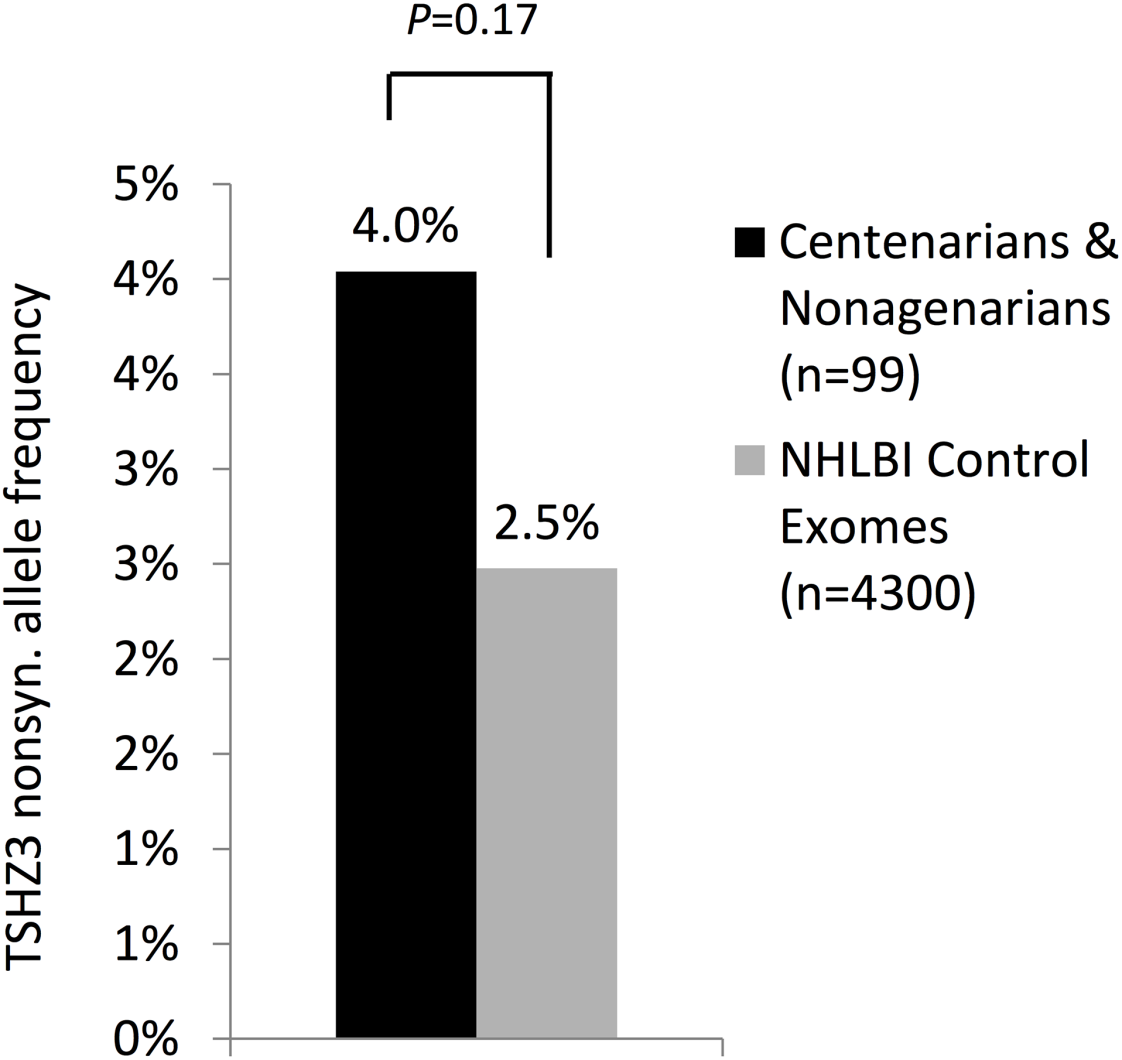
Rare protein-altering variants in TSHZ3 in the Georgia Centenarian cohort versus NHLBI cohort. To see if TSHZ3 is enriched for rare protein-altering variants in long-lived individuals, Sanger sequencing was performed on TSHZ3 in 99 Caucasians with extreme longevity (age 98–105). There was not a significant enrichment comparing the allele frequency of all rare protein-altering variants in the centenarians (4.0%; black bar) to 4300 Caucasian controls from the NHLBI exome project (2.5%; white bar). Both cohorts were annotated for protein-altering variants and filtered against 1000G EUR (MAF<0.015).

To validate the result from the analysis of the supercentenarian genomes, we examined whether TSHZ3 is enriched for rare protein-altering variants in a cohort of 99 people aged 98–105 years from the Georgia Centenarian Study compared to 4,300 control exomes from the NHLBI Exome Variant Server [38] (Table 2). We obtained DNA samples of Caucasian nonagenarians and centenarians and performed Sanger sequencing of the TSHZ3 gene in all long-lived subjects. We used the same filter as for the genome-wide burden test of the supercentenarian genomes (MAF>0.01 in 1000G EUR.). We discovered a higher frequency of protein-altering alleles in the TSHZ3 sequence from 99 long-lived genomes (8 variants; 4%) than in the 4,300 Caucasian controls from the NHLBI cohort (213 variants; 2.5%), but this difference was not statistically significant (P = 0.17; Figure 2; Table 3). Analysis of a larger cohort of supercentenarians may show that the small difference in variants in TSHZ3 compared to controls is statistically significant.

**Table 2:** Baseline statistics of follow-up cohorts.

	Georgia Centenarian	NHLBI
	Study	Controls
Sample size, n	99	4300
Age, mean (range)	101 (98–105)	(≥18)
Females, n (%)	82 (83%)	2428 (56%)

Ages for Georgia Centenarian Study subjects were obtained from Corriell website. Number of females from NHLBI cohort was derived for X chromosome genotypes. Age information for NHLBI controls was obtained from www.nhlbi.nih.gov/recovery/media/NHLBI_DNA_cohort.htm.

**Table 3:** Protein-altering variants in TSHZ3 in Georgia Centenarian cohort.

Position on Chr19	Ref/Var	AA Pos	AA1/AA2	Supercent	Cent	Nona	1000G EUR MAF
31769738	G/A	321	R/W	0	1	0	novel
31769366	C/T	445	V/M	1	0	0	0.0013
31769293	T/C	469	E/G	1	2	1	0.01
31769021	T/C	560	M/V	0	1	0	novel
31768639	G/A	687	P/L	1	0	0	novel
31768594	A/C	702	L/W	0	0	1	novel
31768267	G/A	811	T/M	0	1	0	novel
31768178	C/T	841	E/K	1	0	0	novel
31767599	C/T	1034	E/K	0	1	0	novel

Position (bp) on chromosome 19 (Chr19) of variant, reference (Ref) and Variant (Var) allele, Amino Acid (AA) position, AA1 (ref), AA2 (var), Supercentenarian carriers (shown for reference), Centenarians carriers, Nonagenarians carriers, Minor allele frequency (MAF) in 1000G EUR.

In summary, the results from all three analyses do not show a statistical enrichment for a gene harboring rare protein-altering variants in female Caucasian supercentenarians compared to controls.

### Do Supercentenarians Carry Pathogenic Alleles?

WGS has revealed that seemingly healthy individuals can carry pathogenic mutations that are potentially fatal [50]. Based on their extreme longevity, supercentenarians can be viewed as extremely healthy individuals. We asked whether these extremely healthy individuals might also carry pathogenic mutations. To do this, we analyzed all 17 supercentenarian genomes for the presence of pathogenic alleles as defined by the recent publication of the American College of Medical Genetics and Genomics (ACMG) [39]. The ACMG recommends that these mutations be reported to the patient, even if they are incidental findings. Their paper was a concerted and systematic effort resulting in a list of 56 genes, which are known to harbor strongly pathogenic mutations known to be fatal.

Two supercentenarians possessed a variant that was annotated as being pathogenic by the Human Gene Mutation Database (HGMD) or ClinVar. The first supercentenarian carried a missense SNP (L1564P) in the Breast Cancer Associated 1 (BRCA1) gene. Although null mutations in BRCA1 are pathogenic, the pathogenicity of L1564P is unclear. The L1564P variant appeared in the breast cancer of a 33-year old female along with another missense SNP (Q1785H) [51]. Using an *in vitro* assay, it was found that both missense SNPs in this breast cancer were mild alleles that partially reduced, but did not eliminate, BRCA1 protein function [52]. The L1564P mutation, the Q1785H mutation or both together may have caused breast cancer in this one individual. Hence, the pathogenicity of the L1564P mutation in our supercentenarian remains unclear.

**Figure 3:**
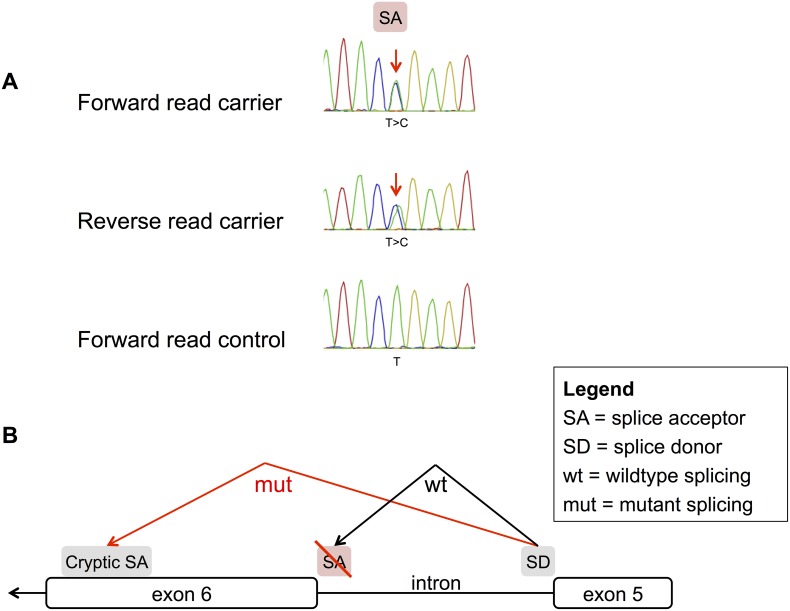
A supercentenarian with a known pathogenic mutation implicated in cardiomyopathy. (A) Sanger validation confirmed that one supercentenarian possessed a known pathogenic mutation in a splice acceptor site of Desmocollin-2 (DSC2), a component of the myocardial desmosome. (B) This rare mutation has been reported in 2 independent cases of Arrhythmogenic Right Ventricular Cardiomyopathy and has been shown to cause cryptic splicing and mRNA degradation [54], [55].

The second supercentenarian possessed a known pathogenic SNP (rs397514042) that disrupts a splice-site in Desmocollin-2 (DSC2). Desmocollin-2 is part of the myocardial desmosome structure in the heart. Loss-of-function mutations in DSC2 and other genes of the desmosome are associated with Arrhythmogenic Right Ventricular Cardiomyopathy (ARVC) [53]. rs397514042 causes an A -> G change in the splice acceptor site of exon 6 of DSC2. Sanger sequencing validated the presence of this SNP in the supercentenarian genome (Figure 3). The variant is annotated as a pathogenic mutation in HGMD, ClinVar, and the Locus Specific Database (LSDB) ARVD/C, which is part of the Leiden Open Variation Database (LOVD).

The rs397514042 SNP has been observed in two patients with ARVC [54], [55]. Heuser et al. further showed that the mutant allele (rs397514042) leads to a decrease in DSC2 mRNA and protein in the patient compared to the reference allele. In zebrafish lacking DSC2, expression of the wild-type human allele rescued the mutant phenotype and led to normal desmosomes, but expression of the mutant human allele corresponding to rs397514042 did not fully rescue the mutant phenotype and resulted in malformed desmosomes. Although the evidence suggests that this SNP can be highly pathogenic, its penetrance is unknown. The supercentenarian subject carrying rs397514042 was asymptomatic to the best of our knowledge and died from a cause unrelated to cardiomyopathy. We conclude that at least 1 out of 17 supercentenarians possessed a known pathogenic SNP.

## Discussion

We have sequenced the genomes of 17 supercentenarians (over 110 years of age) to see if we could uncover the genetic basis for their extreme longevity. We analyzed rare protein-altering variants, but found no strong evidence for enrichment of either a single variant or a single gene harboring different variants in female Caucasian supercentenarians compared to controls. From our gene-based analysis, the gene showing the most enrichment for protein-altering variants in supercentenarians compared to controls was the TSHZ3 transcription-factor gene. Because it was the top hit, we pursued this gene further in a study consisting of 99 genomes from subjects aged 98–105 years old. We found that TSHZ3 carried protein-altering variants in more of the long-lived subjects than the controls, although this difference was not statistically significant (P = 0.17).

A larger sample size would be required to establish whether the difference in frequency of protein-altering variants in TSHZ3 between subjects with extreme longevity compared to controls is statistically significant. We did not analyze single nucleotide variants in non-coding DNA in the supercentenarians because of the large number of non-coding variants compared to coding variants. Our analysis of putative rare protein-altering variants in the whole genome sequencing data led us to test a number of candidates, of which 30% were subsequently determined to be false positive variant calls in WGS data. This high false discovery rate is consistent with previous reports [34] and is largely due to a selection bias as sequencing errors often appear as rare protein-altering variants [33].

Our analyses show that it is extremely unlikely that there is a single gene harboring rare protein-altering variants shared by all supercentenarians but no controls. It is not surprising that a highly complex trait such as longevity is not explained by a single Mendelian gene.

To our surprise, we discovered that one of our supercentenarians carried a known pathogenic allele in the DSC2 gene associated with arrhythmogenic right ventricular cardiomyopathy (ARVC). This is a potentially fatal condition, causing affected individuals to die of sudden cardiac death. This example points out an important aspect about policy regarding the reporting of pathogenic mutations found in genomic sequences. The American College of Medical Genetics and Genomics identified a set of genes that can cause pathology when disrupted. But what is often not known is how frequently people with the variant have pathology (i.e., the penetrance). Our example shows that the DSC2 pathogenic mutation rs397514042 did not cause a fatal cardiomyopathy during the proband’s over 110 years of life. Thus, the presence of this mutation in the DNA sequence of a young person today should be reported to him/her and their families with caution, as it may or may not result in arrhythmogenic right ventricular cardiomyopathy. Generally, variants that are annotated as pathogenic are of unknown penetrance [56].

The full set of protein-coding variants are given in Table S2 and the full-genome sequence from this paper are publicly available via dbGAP and Google Genomics. By making our data available as a public resource, we hope it can be included in future meta-analyses of supercentenarian genomes. Supercentenarians are extremely rare and their genomes could hold secrets for the genetic basis of extreme longevity.

## Supporting Information

Figure S1 **Genome coverage for supercentenarians.** Average genome coverage is shown for the whole genome (dark grey) and exome (light grey) of all 17 supercentenarians. Coverage is shown for ≥1x and ≥20x coverage.
(PDF)

Figure S2 **Principal Component Analysis of supercentenarian ancestry.** PCA was performed on all 17 supercentenarians (black dots) and HapMap genotypes. All Caucasian supercentenarians (CAU) clustered with Caucasian HapMap individuals, while the two supercentenarians of Hispanic ethnicity clustered with Mexican HapMap individuals and the African-American supercentenarian (AA) clustered with African HapMap individuals. HapMap populations are ASW (African ancestry in Southwest USA), CEU (Utah residents with Northern and Western European ancestry from the CEPH collection), CHB (Han Chinese in Beijing, China), CHD (Chinese in Metropolitan Denver, Colorado), GIH (Gujarati Indians in Houston, Texas), JPT (Japanese in Tokyo, Japan), LWK (Luhya in Webuye, Kenya), MXL (Mexican ancestry in Los Angeles, California), MKK (Maasai in Kinyawa, Kenya), TSI (Toscani in Italy) and YRI (Yoruba in Ibadan, Nigeria). See insert for color codes.
(PDF)

Table S1 **Baseline statistics for 34 Caucasian PGP genomes.**
(XLSX)

Table S2 **All variants in protein coding regions with genotypes for all 17 supercentenarian and 34 PGP control genomes.**
(XLSX)

Table S3 **Burden of rare protein-altering variants per gene in supercentenarians and controls.**
(XLSX)

Table S4 **Gene-based Fisher’s Exact test for recessive model of rare protein-altering variants in supercentenarians and controls.**
(XLSX)
