# Supplementary material for: Whole-Genome Sequencing of the World’s Oldest People
Source: PLoS One. 2014 Nov 12;9(11):e112430. doi: 10.1371/journal.pone.0112430 (PMC4229186; doi:10.1371/journal.pone.0112430)
Supplement: Figure S1 — Genome coverage for supercentenarians. Average genome coverage is shown for the whole genome (dark grey) and exome (light grey) of all 17 supercentenarians. Coverage is shown for ≥1x and ≥20x coverage. (PDF) [file pone.0112430.s001.pdf]

Figure S1

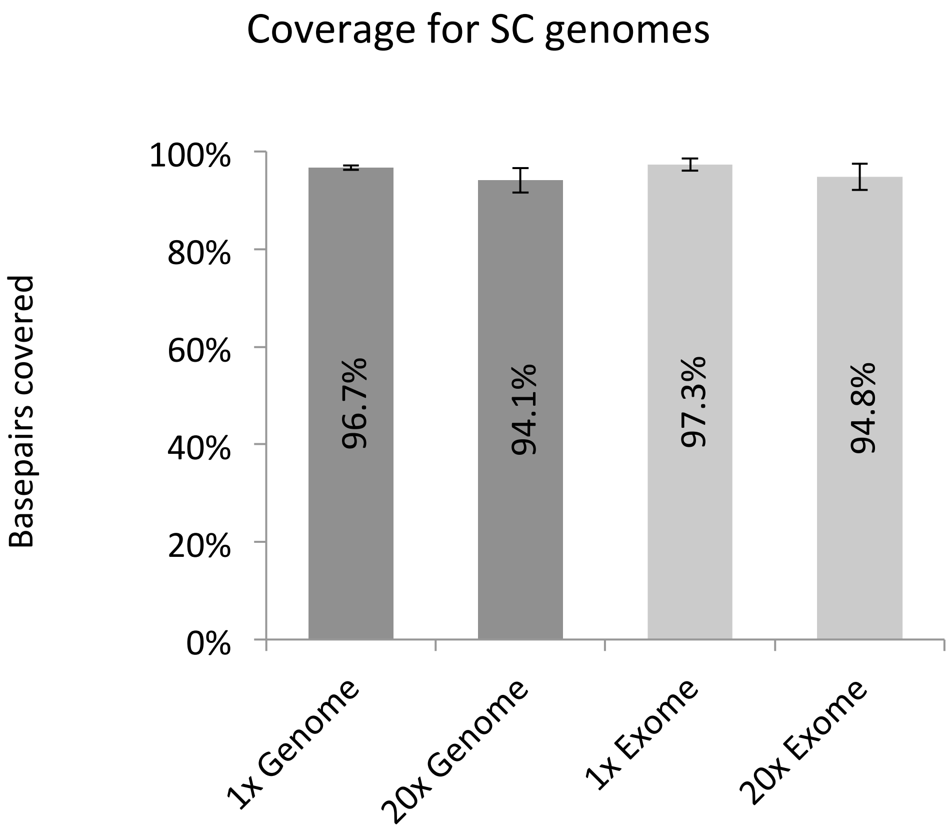

Figure S1 Genome coverage for supercentenarians. Average genome coverage is shown for the whole genome (dark grey) and exome (light grey) of all 17 supercentenarians. Coverage is shown for ≥1x and ≥20x coverage.
