# Supplementary material for: Whole-Genome Sequencing of the World’s Oldest People
Source: PLoS One. 2014 Nov 12;9(11):e112430. doi: 10.1371/journal.pone.0112430 (PMC4229186; doi:10.1371/journal.pone.0112430)
Supplement: Figure S2 — Principal Component Analysis of supercentenarian ancestry. PCA was performed on all 17 supercentenarians (black dots) and HapMap genotypes. All Caucasian supercentenarians (CAU) clustered with Caucasian HapMap individuals, while the two supercentenarians of Hispanic ethnicity clustered with Mexican HapMap individuals and the African-American supercentenarian (AA) clustered with African HapMap individuals. HapMap populations are ASW (African ancestry in Southwest USA), CEU (Utah residents with Northern and Western European ancestry from the CEPH collection), CHB (Han Chinese in Beijing, China), CHD (Chinese in Metropolitan Denver, Colorado), GIH (Gujarati Indians in Houston, Texas), JPT (Japanese in Tokyo, Japan), LWK (Luhya in Webuye, Kenya), MXL (Mexican ancestry in Los Angeles, California), MKK (Maasai in Kinyawa, Kenya), TSI (Toscani in Italy) and YRI (Yoruba in Ibadan, Nigeria). See insert for color codes. (PDF) [file pone.0112430.s002.pdf]

Figure S2

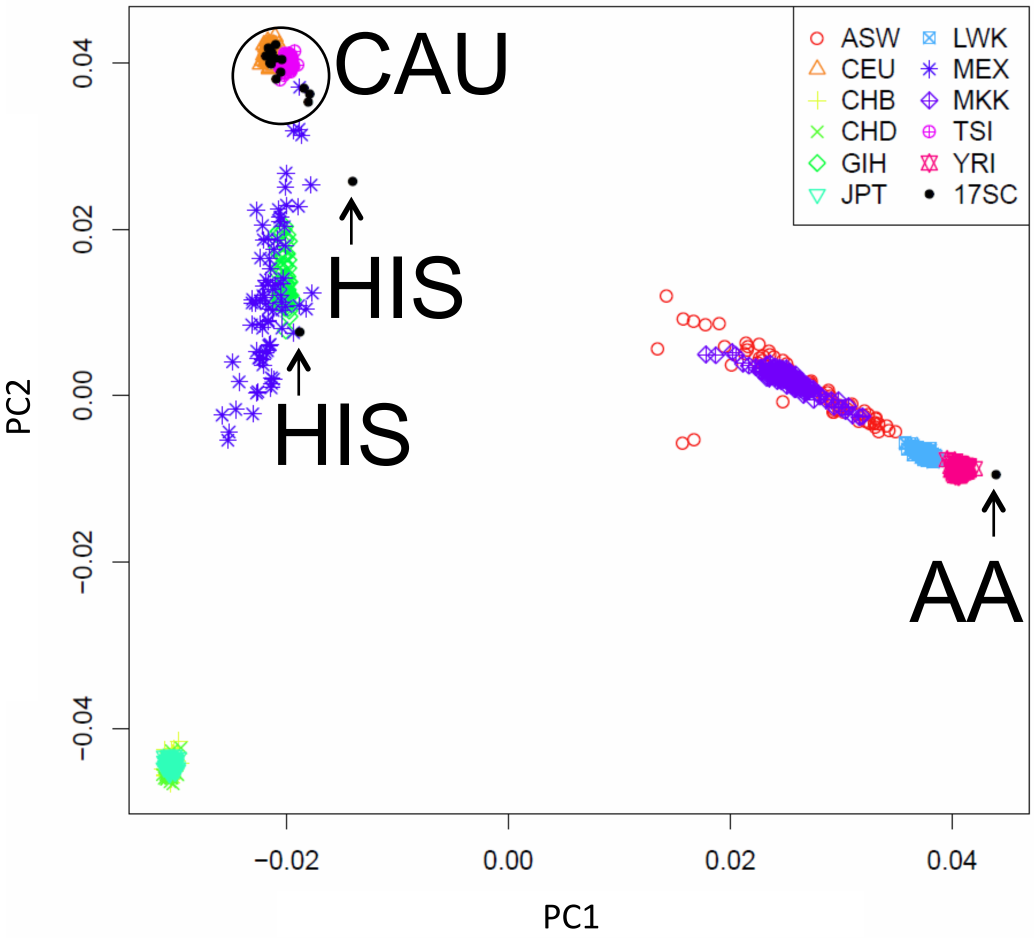

Figure S2 Principal Component Analysis (PCA) of supercentenarian ancestry. PCA was performed on all 17 supercentenarians (black dots) and HapMap genotypes. All Caucasian supercentenarians (CAU) clustered with Caucasian HapMap individuals, while the two supercentenarians of Hispanic ethnicity clustered with Mexican HapMap individuals and the African-American supercentenarian (AA) clustered with African HapMap individuals. HapMap populations are ASW (African ancestry in Southwest USA), CEU (Utah residents with Northern and Western European ancestry from the CEPH collection), CHB (Han Chinese in Beijing, China), CHD (Chinese in Metropolitan Denver, Colorado), GIH (Gujarati Indians in Houston, Texas), JPT (Japanese in Tokyo, Japan), LWK (Luhya in Webuye, Kenya), MXL (Mexican ancestry in Los Angeles, California), MKK (Maasai in Kinyawa, Kenya), TSI (Toscani in Italy) and YRI (Yoruba in Ibadan, Nigeria). See insert for color codes.
